# Are lipid nanoparticles really superior? A holistic proof of concept study

**DOI:** 10.1007/s13346-021-01021-5

**Published:** 2021-09-03

**Authors:** Sabrina Wiemann, Cornelia M. Keck

**Affiliations:** Department of Pharmaceutics and Biopharmaceutics, Philipps-Universität Marburg, Robert-Koch-Str. 4, 35037 Marburg, Germany

**Keywords:** Dermal drug delivery, Skin care, Lipid nanoparticles, Nanostructured lipid carriers, Nanoemulsion, Occlusion

## Abstract

Lipid nanoparticles are a successful carrier system for dermal drug delivery. They possess various beneficial properties, i.e., increased chemical stability for chemically labile compounds, increased dermal penetration of active compounds, or skin carrying properties after dermal application due to the formation of a so-called “invisible patch.” Despite manifold studies showing these properties individually, a study that investigates if one lipid nanoparticle formulation can really combine all the above-mentioned benefits at once is not yet available. In the present study, lipid nanoparticles (NLC) were produced and characterized regarding their physico-chemical properties. The chemical stability of the incorporated active ingredient (AI) was determined, as well as the dermal penetration efficacy of the AI, and the skin carrying properties of the NLC after dermal penetration. The properties of the NLC were compared to classical formulations, i.e., AI dissolved in pure oil, an o/w cream base and a nanoemulsion. All formulations contained similar lipids and emulsifiers, which allowed for a direct comparison of the different properties. NLC were shown to provide most efficient chemical stabilization and most efficient dermal penetration for the AI. The formation of the invisible patch was shown for the NLC but not for the other formulations. Skin hydration and skin carrying properties were also most pronounced for the NLC. Results provide evidence that NLC can combine all beneficial effects that were previously described in one formulation. Thus, providing evidence that NLC are a holistically superior formulation principle when compared to other formulation principles.

**Graphical abstract:**

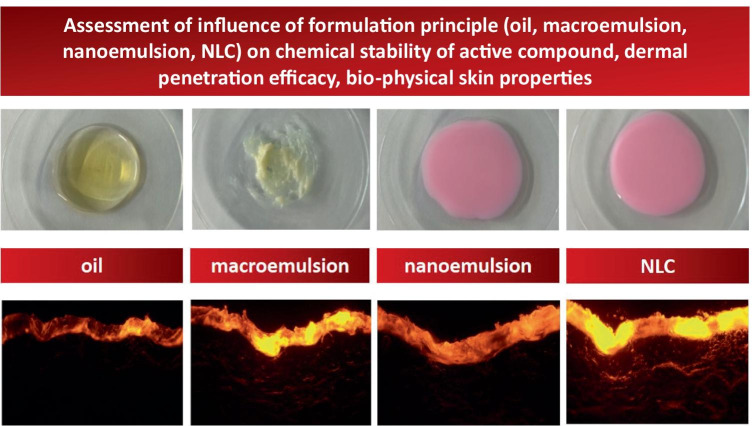

**Supplementary Information:**

The online version contains supplementary material available at 10.1007/s13346-021-01021-5.

## Introduction

**Fig. 1:**
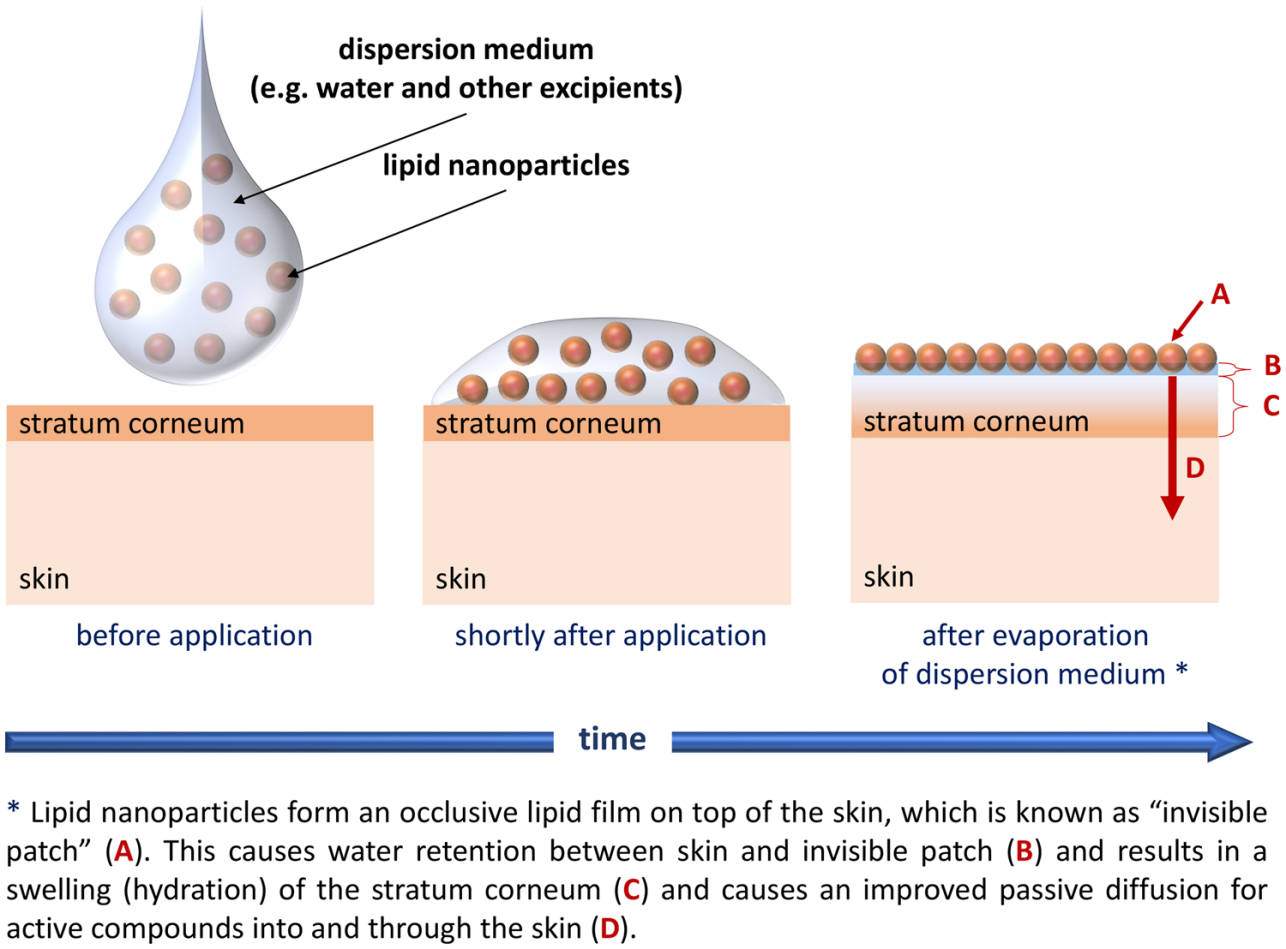
Scheme of invisible patch that is formed after dermal application of LN

Lipid nanoparticles are well-recognized drug delivery systems that were independently invented by Maria Rosa Gasco (Turin/Italy) and Rainer H. Müller (Berlin/Germany) in the early 1990ties. Lipid nanoparticles are characterized by a solid lipid matrix in which lipophilic active ingredients (AI) can be encapsulated. The encapsulation of AI into lipid nanoparticles (LN) increases the chemical stability of chemically labile AI [1]. After dermal application, the LN form a lipid film which is known as “invisible patch” [2]. The invisible patch re-enforces the natural lipid film of the stratum corneum (SC), increases skin hydration, and increases the dermal penetration of lipophilic AI (Fig. 1). Three different generations of lipid nanoparticles (SLN^®^, NLC^®^, and smartLipids^®^) are known today, and manyfold scientific studies could already prove the superiority of lipid nanoparticles over other formulation strategies [3–13]. The lipid nanoparticles are exploited in various cosmeceutical products, and very recently, they were even described to be the most successful carrier for skin delivery [14].

Based on the massive number of scientific studies and products on the market, one would expect that a final proof of concept study showing that lipid nanoparticles are indeed superior in all above-mentioned aspects when compared to classical formulations, i.e., emulsions or creams, or to nanoemulsions (NE), which are much easier to develop, has long been conducted. Interestingly, based on our findings, this is not the case. Many studies investigate only one aspect, e.g., chemical stability of the AI, dermal penetration efficacy, or skin carrying properties. In many cases, the efficacy of lipid nanoparticles is only compared to one other drug delivery system and/or to formulations that contain other ingredients than the lipid nanoparticles. Hence, a comprehensive study that compares the efficacy of lipid nanoparticles in regard to all their beneficial properties to different formulation principles is not available today. This means a holistic study that investigates if lipid nanoparticles can really combine all these beneficial properties in only one formulation is not available.

Therefore, the aim of this study was to produce lipid nanoparticles and to compare their properties to other formulation principles (oil, macroemulsion (ME), NE) that contained similar excipients as the lipid nanoparticles. The similarity in chemical composition of the formulations allowed a direct comparison of the properties, i.e., chemical stability of the incorporated AI, dermal penetration efficacy, and skin carrying properties, between the different formulations.

## Materials and methods

### Materials

The lipophilic fluorescent dye 1,1′-dioctadecyl-3,3,3′,3′-tetramethylindo-carbocyanin perchlorate (DiI perchlorate, Biozol Diagnistica Vertrieb GmbH, Germany) was chosen as a surrogate of an incorporated lipophilic AI. All formulations contained 0.005% (w/w) DiI. This concentration was chosen, because it enabled a sufficient autofluorescence for the skin penetration experiments and ensured an efficient encapsulation of the dye into the lipid phase without expulsion into the water phase at the same time. Sea fish oil (SFO) was selected as omega-3-rich liquid lipid source and was kindly provided by San Omega GmbH, Germany. Glyceryl tristearate (Dynasan^®^ 118, kindly provided by IOI Oleo GmbH, Germany) was used as solid lipid and structure-forming component for the o/w cream (macroemulsion, ME) and the NLC. The formulations were stabilized by using a blend of four non-ionic surfactants, i.e., Poloxamer 188 (PLX 188, kindly provided by BASF, Germany), Polysorbate 80 (Tween^®^ 80, VWR, Germany), Polyvinylpyrrolidone 10,000 (PVP, AppliChem GmbH, Germany), and d-α-tocopheryl polyethylene glycol 1,000 succinate (TPGS, Antares Health Products Inc., USA). The selection was based on a previous study by Muchow et al. [15]. Purified water was used as dispersion medium and was freshly obtained from a PURELAB Flex 2 (ELGA LabWater, Veolia Water Technologies Deutschland GmbH, Germany). Unless otherwise specified, all other chemicals were used as received. Table 1 provides an overview of the compositions of the formulations that were produced and characterized in this study.

**Table 1:** Composition of the oily solution (Oil), macroemulsion (ME), nanoemulsion (NE), and nanostructured lipid carriers (NLC) in % (w/w)

	Oil	ME	NE	NLC
DiI perchlorate	0.005%	0.005%	0.005%	0.005%
Sea fish oil (SFO)	99.995%	55.995%	9.995%	3.995%
Glyceryl tristearate	-	14.0%	-	6.0%
Aqueous phase*	-	30.0%	90.0%	90.0%

* The aqueous phase consisted of Poloxamer 188 (3.0%), Tween 80 (0.1%), PVP (0.1%), TPGS (1.0%), and purified water (ad 100.0%)

### Methods

The study was divided into two parts. In the first part, ME, NE, and NLC were produced and characterized regarding their physico-chemical properties. In the second part, their biopharmaceutical properties (ex vivo dermal penetration efficacy, influence on bio-physical skin parameters) were determined and compared to an oily solution (Table 1).

#### Production of the formulations

The oil formulation was produced by dissolving the dye in sea fish oil (SFO). ME, NE, and NLC were produced after a previously established protocol [15]. First, DiI perchlorate (0.005%_(w/w)_) was dissolved in the SFO. In the case of the ME and NLC, the solid lipid (Dynasan 188) was added. Next, the lipid phase of each colloidal formulation was heated to 70 °C. For the aqueous phase, PLX 188, Tween 80, PVP, and TPGS were dissolved in purified water and likewise heated. Afterwards, the hot aqueous phase was slowly poured into the lipid phase while constant stirring. The obtained mixture was high speed stirred for 30 s at 10,000 rpm by using an Ultra Turrax T25 (IKA-Werke GmbH & Co. KG, Germany). The pre-emulsion was then subjected to hot high-pressure homogenization (HPH) via a LAB 40 (APV Gaulin, Germany) by applying 3 cycles at 500 bar in discontinuous mode. The production temperature was set to 70 °C. The obtained formulations were immediately cooled in an ice bath and stored in falcon tubes (50 mL) at room temperature until further use.

#### Physico-chemical characterization

### Size characterization

Particle sizes were analyzed by three independent measuring principles: dynamic light scattering (DLS), laser diffraction (LD), and light microscopy. DLS, also known as photon correlation spectroscopy (PCS), was used to examine the hydrodynamic particle diameter (z-average) and the polydispersity index (PdI) of the produced formulations. The measurements were conducted with a Zetasizer Nano ZS (Malvern Panalytical Ltd, UK). Measurements were performed at 20 °C and were analyzed with the general purpose mode built in into the software of the instrument. LD was used to detect possible larger particles, since the measuring range of the Mastersizer 3000 (Malvern Panalytical Ltd, UK) covers sizes up to 3 mm (3000 µm). Data analysis was performed by using Mie theory. The real refractive index was set to 1.5 and the imaginary refractive index to 0.001. Size and size distribution obtained are expressed as median volumetric particle diameters (d(v)x [µm]) [16]. The formulations were characterized on the day of production and during storage over 35 days at room temperature. Light microscopy was performed to confirm the results obtained from LD [17] and by using an Olympus BX53 light microscope (Olympus Corporation, Japan), equipped with an Olympus SC50 CMOS color camera (Olympus soft images solution GmbH, Germany). In addition, to gain a more detailed knowledge on the colloidal structure of the produced formulations, their aqueous and lipophilic phases were visualized with methylene blue (hydrophilic dye) and sudan (III) red (lipophilic dye). For this approximately 0.5 g of each formulation were placed on watch glass dishes and approximately 10 mg of each dye reagent was blended with each sample. After 5 min incubation period, the formulations were analyzed by light microscopy.

### Zeta potential analysis

The zeta potential (ZP) was determined by measuring the electrophoretic mobility (EM) via laser Doppler anemometry (Zetasizer Nano ZS, Malvern Panalytical Ltd, UK). The samples were prepared for analysis by dispersing the produced formulations in original dispersion medium (i.e., aqueous phase; Table 1). Additionally, particles were also dispersed in purified water with adjusted conductivity (50 µS/cm). The analyses were carried out at 20 °C, and the EM was converted into the ZP by using the Helmholtz-Smoluchowski equation [18].

### Chemical stability

DiI is a chemically labile compound that changes its color upon oxidation from magenta to yellow. The chemical stability was therefore determined by observing and comparing the changes in color from the different formulations over time. For this purpose, approx. 1 mL of each formulation, i.e., oily solution and colloidal systems, was placed on a watch glass. Images of the formulations were taken immediately after sampling and after storage in an oven for 6 h at 32 °C. The storage conditions of the formulations were chosen to mimic the skin penetration experiments and were therefore set as minimum requirements for chemical stability of the surrogate AI.

#### Biopharmaceutical characterization

The biopharmaceutical properties were assessed by determining the dermal penetration efficacy of the dye from the different formulations and their influence on the bio-physical skin properties, i.e., skin hydration, transepidermal water loss (TEWL), and skin friction.

### Determination of dermal penetration efficacy

The dermal penetration efficacy was determined on the ex vivo porcine ear model. Fresh porcine ears were obtained from a local slaughterhouse, and the experiments were conducted on the day of slaughter. The ears were washed with lukewarm water (approximately 23–25 °C) and gently dried with paper towels in dabbing movements without rubbing. Examination areas of 2 × 2 cm with no injuries were defined on the dorsal side of the ears. The hairs in these areas were carefully cut to a length of about 1–3 mm. Afterwards, 50 μL of each formulation were applied on the examination areas and massaged into the skin for 30 s using the saturated glove method [19]. Skin penetration was conducted for 1 h and 6 h at 32 °C. Afterwards, the skin surface was carefully wiped with wet paper towels to remove any sample residues on the skin surface. Subsequently, punch biopsies with a diameter of 15 mm were taken, embedded in Tissue-Tek^®^ (Sakura Finetek Europe B.V., Netherlands) and immediately frozen at − 80 °C. Cryo-sectioning in 20 µm thick vertical cuts was performed with a cryomicrotome (Mod. 2700 Reichert-Jung, Germany). Images of skin sections obtained were taken by using an inverted epifluorescence microscope (Olympus CKX53) equipped with an Olympus DP22 color camera and an Olympus U-HGLGPS light source (Olympus Corporation, Japan). The intensity of the fluorescence light source was set to 100%, and the exposure time was set to 50 ms. The filter selected for analysis was the DAPI HC filter block system (excitation filter 540–560 nm, dichroic mirror 570 nm, emission filter starting at 580 nm (LP)). Each formulation was tested in triplicate, i.e., on 3 different, independent porcine ears. From each skin area, at least 12 skin sections were obtained, and from each skin section, at least 3 images were obtained. Hence, for each skin area tested, at least 36 images were obtained, which resulted in a total of at least 108 images for each sample (n = 3). These images were used for subsequent digital image analysis.

### Digital image analysis

Digital image analysis was performed with “ImageJ” software [20, 21], and the epifluorescence images with 200-fold magnification were used for this. The penetration efficacy was assessed by determination of the total amount of penetrated dye (TAP) and by assessing the mean penetration depth (MPD) [22]. TAP and MPD were determined from the images after subjecting each image to an automated threshold algorithm (supplementary material Sect. 1) to eliminate the autofluorescence of the skin [22]. The remaining light pixels corresponded to the dye that penetrated the skin. The TAP was determined as mean grey value/pixel within each image, and the MPD was determined by measuring the distance between SC surface and penetrated dye [22]. The hydration of the stratum corneum (SC) was also assessed by measuring the SC thicknesses (SCT) from the original non-processed images. The scale bar of the images corresponded to 50 µm and was set to 142 pixels in the software. The SCT was converted into the relative SCT by comparing the measured SCT of the treated skin to the SCT of untreated skin, which was set to 100%.

### Determination of bio-physical skin parameters

The influence of the formulations on the bio-physical skin properties, i.e., barrier protection, film formation, and skin hydration, were investigated by measuring transepidermal water loss (TEWL), skin hydration, and skin friction of the porcine skin. The parameters were assessed prior to the application of the formulations and 1 h and 6 h after application. Prior to the measurements, excess formulation was carefully removed with a dry paper towel in dabbing movements. Measurements were performed with appropriate skin probes (Tewameter^®^ TM300 for TEWL, Corneometer^®^ CM825 for skin hydration and Frictiometer^®^ FR700 for skin friction). The probes were kindly provided by Courage & Khazaka Electronic GmbH, Germany. Experiments were performed in triplicate, i.e., on three different, independent porcine ears.

#### Statistical analysis

Statistical analysis was performed with JASP software (Version 0.14.1). Normal distribution and variance homogeneity of the data were tested with the Shapiro–Wilk and the Levene’s test, respectively. For the normally distributed data, the mean values were compared by a one-way ANOVA, that was Welch-adopted in case of variance heterogeneity. Tukey or Games-Howell post hoc tests were performed to compare the mean values between each other. For the non-parametric data sets, a Kruskal–Wallis analysis of variance, followed by Dunn`s post hoc tests, was conducted [23]. P < 0.05 were considered statistically significant.

## Results and discussion

### Physico-chemical properties of the formulations

**Fig. 2:**
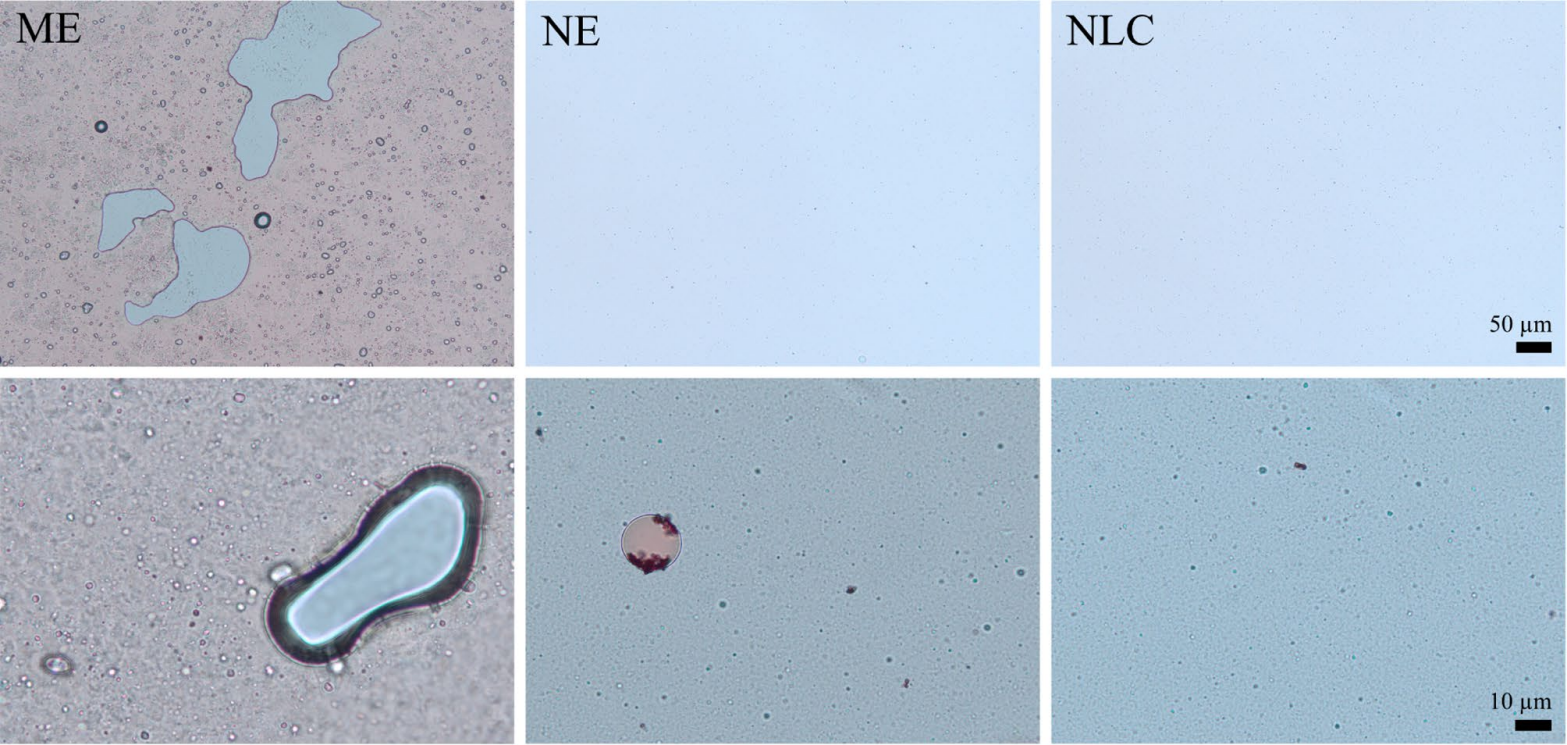
Light microscopic images of the formulations after staining with methylene blue and sudan (III) red. Upper: 200-fold magnification, lower: 1000-fold magnification

The macroemulsion (ME) obtained was a semi-solid formulation with a mean droplet size of about 10–15 µm (Fig. 2; Table 2). Despite the large droplets, the formulation also contained small-sized droplets with sizes in the range of about 320 nm (PCS data). The colloidal structure of the ME was found to be not a simple, disperse system that represented an oil in water or water in oil emulsion but was rather a bi-coherent system that consisted of oil and water gel phases in which larger water droplets and small-sized lipid particles were dispersed (Fig. 2).

**Table 2:** Particle size analysis (PCS and LD) and zeta potentials (ZP—measured in original dispersion medium (OM) and water) of the formulations immediately after production. Mean values ± SD. For physical stability data—cf. supplementary material Sect. 2

		ME	NE	NLC
PCS	z-average (nm)	317 ± 71	151 ± 2	166 ± 5
	PdI	0.44 ± 0.20	0.13 ± 0.04	0.13 ± 0.04
LD (µm)	d(v)0.5	13 ± 0.98	0.08 ± 0.00	0.08 ± 0.00
	d(v)0.9	42 ± 2.56	0.26 ± 0.00	0.27 ± 0.00
	d(v)0.95	51 ± 3.21	0.34 ± 0.00	0.36 ± 0.00
	d(v)0.99	69 ± 4.49	0.5 ± 0.00	0.6 ± 0.00
ZP (mV)	in OM	− 3.0 ± 0.48	− 2.1 ± 0.54	− 1.5 ± 0.43
	in water	− 12.7 ± 2.29	− 6.6 ± 0.89	− 5.5 ± 1.20

With this the ME possessed a colloidal structure being comparable to the structure described by H. Junginger for o/w creams. He found that o/w creams with crystalline gel structures, i.e., the water containing hydrophilic ointment (German pharmacopoeia, DAB 8), non-ionic hydrophilic ointment (German drug product codex, DAC), and a stearate cream, do not contain finely dispersed oil droplets in an aqueous, continuous phase. Instead, they rather form a hydrophilic and lipophilic gel phase, which exist as a coherent system in which coarse bulk water is immobilized [24]. Hence, the ME produced possessed a colloidal structure being comparable to the colloidal structure of o/w ointments that are often used as base for the production of prescription dermal formulations in today’s compounding pharmacies.

The NE was an o/w emulsion with droplet sizes of about 150 nm (PCS data; Table 2). The formulation contained some larger sized droplets > 10 µm (Fig. 2), which could not be detected by LD analysis (Table 2). The reason for overlooking larger droplets in emulsions with a nanosized main droplet population is either due to the very low number of larger particles (< 1%) within the emulsion and/or due to the experimental setup of the LD instrument, e.g., the stirrer of the LD instrument that can destroy large oil droplets during the measurement [25]. The NLC possessed a slightly larger particle PCS size of about 165 nm (Table 2), but light microscopy showed the absence of the larger sized droplets found for the NE (Fig. 2). The presence of larger sized particles and a broader size distribution typically results in less physically stable systems. Therefore, the NLC were found to be the most stable formulation during storage (no changes in size during 5 weeks of storage), followed by the NE (small increase in size after 7 days of storage) and the ME (pronounced changes in size during storage—cf. supplementary material Sect. 2).

The differences in physical stability can also be seen in the different results obtained from ZP analysis (Table 2). The blend of non-ionic surfactants used for all formulations should lead to a thick steric layer of surfactant around the particles and thus should lead to low ZP values being close to 0 mV, when measured in the original dispersion medium (OM). If the surfactant layer is tightly bound to the surface of the particles, the ZP should not change when the particles are diluted and analyzed in water. Hence, a high ZP and/or a pronounced difference between the ZP measured in OM and water indicates less efficient steric stabilization and a less tightly bound layer that can be easily washed off upon dilution of the particles with water [18, 26, 27]. The highest ZP and the most pronounced difference between the ZP in water and OM was found for the ME, followed by the NE. The smallest ZP and the smallest difference between ZP_OM_ and ZP_water_ was found for the NLC. Thus, fully supporting the findings from particle size analysis.

#### Chemical stability

**Fig. 3:**
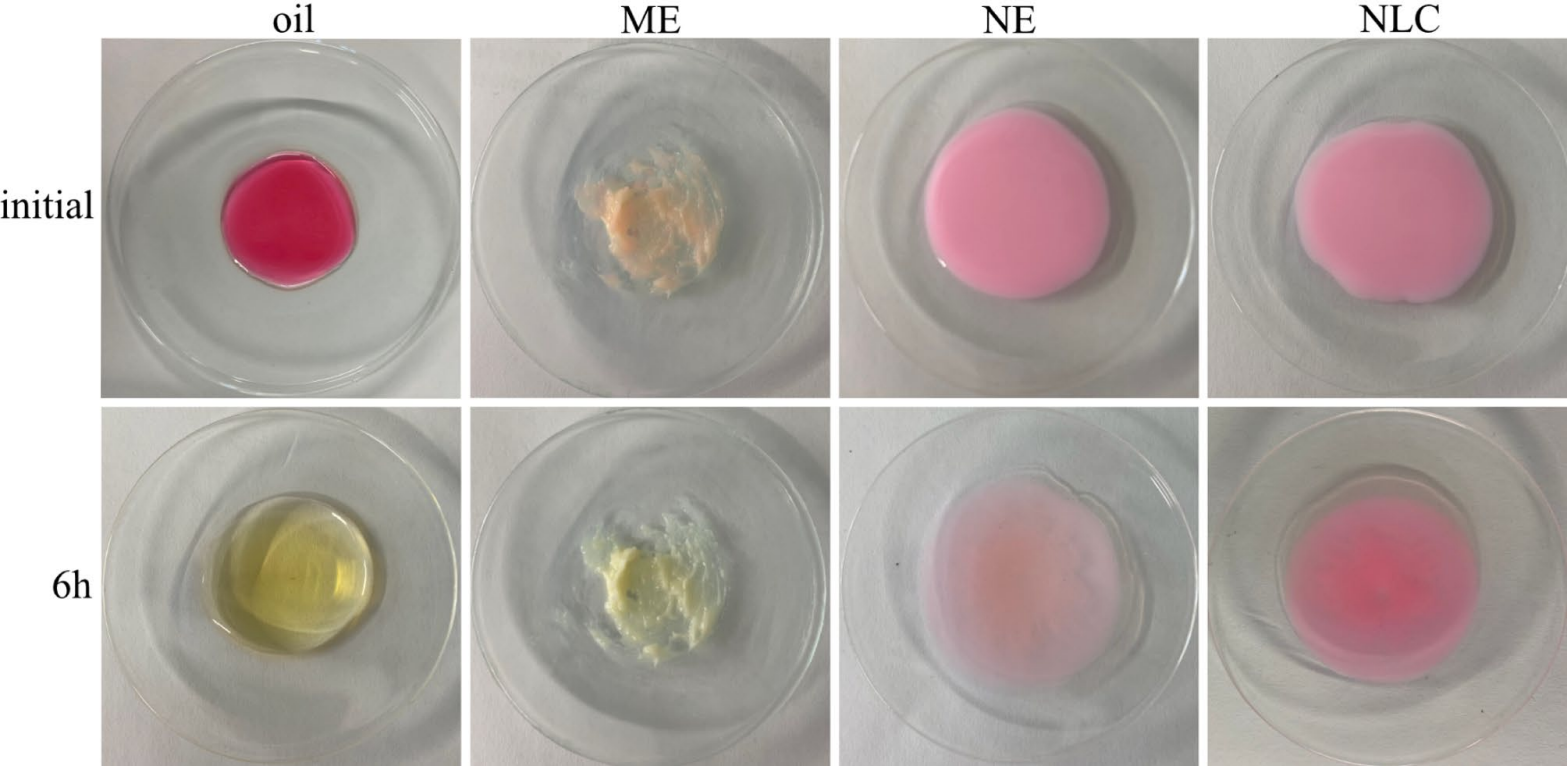
Macroscopic images from stability testing—initial and after 6 h at 32 °C, showing a fast change in color for the oil and the ME (= instability), little change for the NE and no change for the NLC (= stable)

The chemical stability of the formulations followed the same trend (Fig. 3). A very fast change in color, indicating oxidation and thus chemical degradation of the AI surrogate, was observed when the dye was dissolved in oil and stored at 32 °C for 6 h. The loading of the dye in the ME already resulted in a pronounced change in color, indicating that the production of the dye-loaded ME led to a chemical degradation of the AI surrogate. In contrast, no changes in color were observed for the NE and the NLC (Fig. 3—upper). After 6 h of storage, the color of AI surrogate was completely changed when it was dissolved in oil and formulated as ME. A slight change in color was seen for the NE, and no changes were observed for the NLC (Fig. 3—lower). Data indicate that the chemically labile AI surrogate was not protected from oxidation when dissolved in oil or formulated as ME. The AI surrogate was slightly protected when formulated as NE but was most protected when formulated as NLC. Results of this study therefore fully support multiple previous findings, where an increased chemical stability of AI was shown for NLC and explained by a firm incorporation of the AI in the solid lipid matrix of the lipid nanoparticles [1, 4, 11, 28–34].

### Biopharmaceutical properties

#### Dermal penetration efficacy

**Fig. 4:**
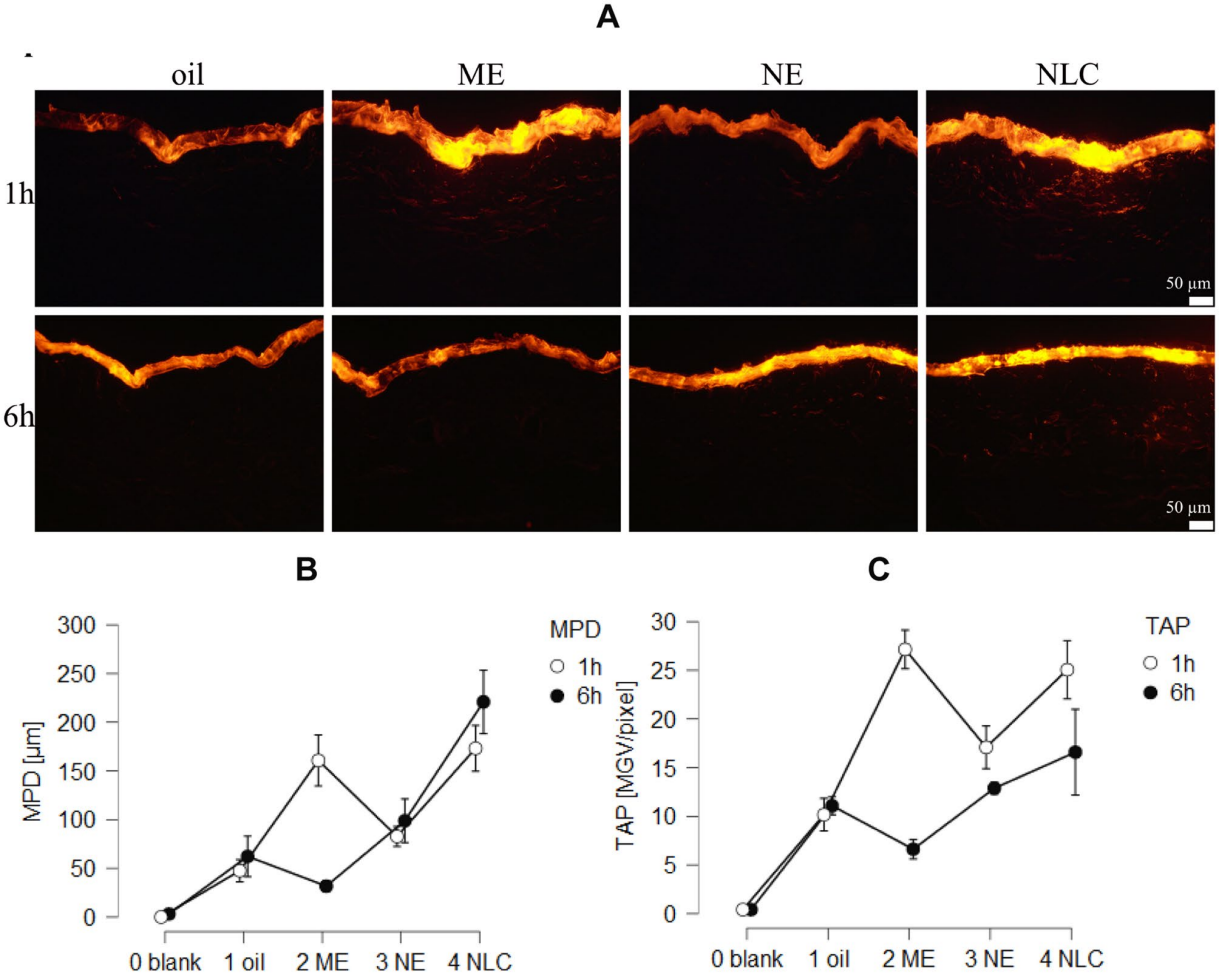
Dermal penetration efficacy of AI surrogate DiI from different formulations (oil, ME, NE and NLC) after 1 and 6 h penetration time. **A** Microscopic images obtained from epifluorescence microscopy (200-fold magnification—detailed microscopic images—cf. supplementary material Sect. 3). **B** Mean penetration depth (MPD). **C** Total amount of penetrated dye (TAP)

**Fig. 5:**
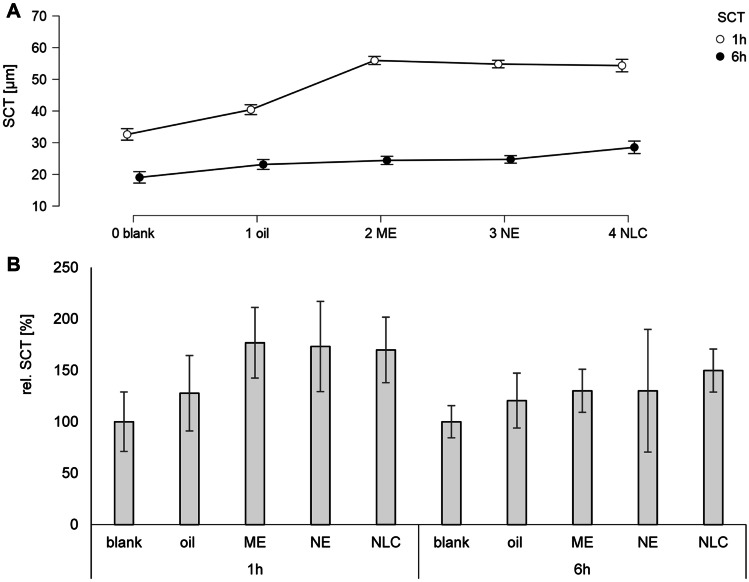
Influence of formulation principle and penetration time on skin hydration. Data represent **A** the absolute stratum corneum thickness (SCT) and **B** the relative stratum corneum thickness (rel. SCT) 1 h and 6 h after application

The penetration efficacy depended on the type of formulation and on the penetration time (Fig. 4). After 1 h penetration time, the most effective penetration of the AI surrogate was found for the ME and the NLC. Both formulations enabled not only a pronounced penetration of the dye into the stratum corneum but also into the viable dermis, i.e., MPD values > 50 µm. The penetration was less efficient from the NE and least efficient from the pure oil. With increasing time, the penetration efficacy increased for the NE and became less efficient for the ME. After 6 h penetration time, the NLC remained most efficient and resulted in the highest TAP and MPD. The NE was the second-best formulation, oil the third-best, and the ME the least efficient formulation (Fig. 4B/C). Results indicate that non-linear and different parameters contribute to the amount of penetrated AI into or through the skin. One major parameter is the chemical stability of the AI in the formulation. DiI, when formulated in oil or the ME, degraded completely within 6 h, which hampered the penetration of intact DiI into the skin from the oil and the ME. Another parameter is skin hydration, which is known to increase the dermal penetration efficacy of AI [35]. Skin hydration is associated with a swelling of the SC that causes an increase in intercellular space between the corneocytes, thus, increasing the diffusion coefficient for the AI into the skin. A swelling of the SC was also observed upon the treatment with the different formulations (Figs. 4A and 5).

The most pronounced swelling of the SC was observed 1 h after application which declined over time. This indicates that water hydrates the skin immediately after application of the formulations but evaporates over time, thus, leading to a decrease in SCT with on-going penetration time (Fig. 5A). The application of oil increased the SCT by about 30% (Fig. 5B). The formulation contained no water. Hence, the increase is due to occlusion, i.e., the water in the skin was prevented from evaporating, thus causing the SC to swell [36, 37]. The ME, NE, and NLC increased the relative SCT to about 175%. The twofold increase in SCT after the application of ME, NE, and NLC when compared to the pure oil indicates that the water (and surfactant, cf. Table 1) containing formulations cause a swelling of the SC not only due to occlusion but also by a direct hydration of the SC with water from the formulations. The hydrating effect is lost when the water evaporates which results in a decrease in SCT after 6 h.

After 6 h of incubation, the occlusive effect of the oil formulation is still visible (approximately + 20% when compared to untreated skin; Fig. 5B) but is higher for the ME and the NE (approximately + 30% when compared to untreated skin; Fig. 5B). The SCT of the skin treated with the NLC was still thicker (approximately + 50% when compared to untreated skin; Fig. 5B), indicating that the NLC formulation was most effective in retaining the water in the SC.

#### Influence of formulations on bio-physical skin properties

**Fig. 6:**
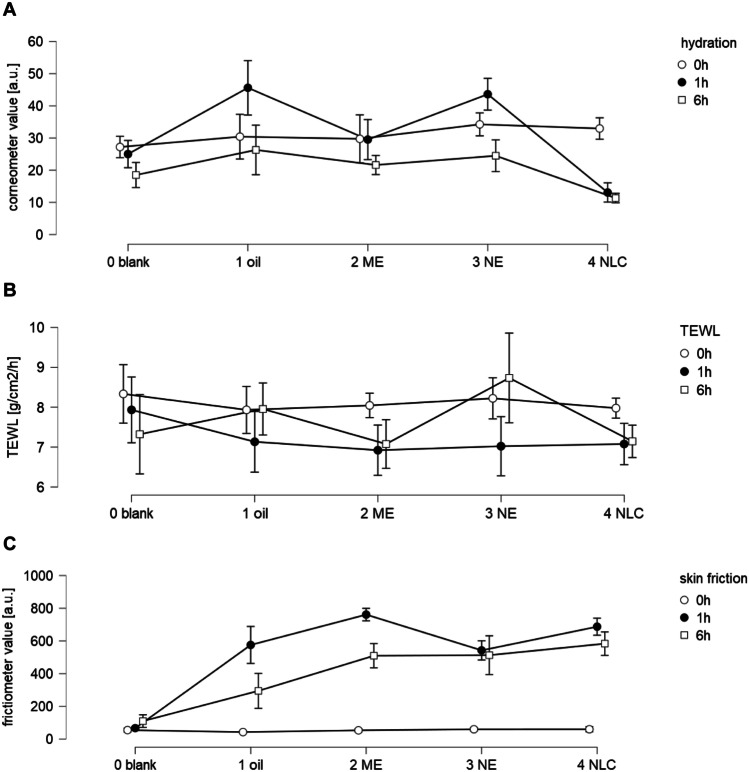
Influence of formulations on bio-physical skin parameters. **A** skin hydration, **B** TEWL, **C** skin friction. Measurements were taken prior to application and after 1 and 6 h after application. Excess formulations were wiped off prior to the measurements

Film formation of formulations on top of the skin causes not only a swelling of the SC but can also change the bio-physical skin properties, e.g., skin hydration, TEWL, or skin friction [32, 37, 38]. Therefore, to gain further knowledge on the skin effects of the different formulations, these properties were also assessed (Fig. 6).

### Skin hydration

The skin hydration was determined by using a Corneometer. The probe determines the capacitance of the SC. High corneometer values typically correspond to a high skin hydration and vice versa [38], but previous studies already showed that NLC upon dermal application can form an invisible lipid film on top of the skin. Consequently, as lipids possess a low dielectric constant, NLC formulations that form such a film on top of the skin result in low corneometer values [2]. In this study, the skin hydration was increased after the application of the oil and the NE 1 h after application. No changes in skin hydration were found for the ME and a decrease in corneometer values was seen for the NLC (Fig. 6A). The decrease in corneometer values for the NLC confirms the formation of the invisible patch, that was previously reported for various other NLC formulations [1, 2, 39, 40]. Six hour after application, no significant differences in skin hydration were found between oil, ME, and NE. The corneometer values of the NLC however were significantly lower, indicating that the invisible patch was still present 6 h after the application of the NLC but not for the other formulations tested.

### TEWL measurements

The TEWL represents the water evaporation rate from the skin in g/cm^2^/h and is a measure of the skin’s barrier function [38]. High TEWL values indicate an increased water loss and thus an impaired skin barrier. A decreased TEWL after application of a formulation indicates a barrier protecting effect of the formulation. Barrier protecting effects, i.e., slightly decreased TEWL values when compared to the untreated skin, were found for all formulations 1 h after application (Fig. 6B). The effect lasted for 6 h for the ME and the NLC but was not seen for the oil and the NE. Hence, long-lasting barrier protecting properties were only found for the ME and the NLC but not for the oil and the NE. After 6 h, the TEWL of the untreated skin was decreased, indicating—similar to the skin hydration measurements (Fig. 6A)—a slight dehydration of the skin during the experiment.

### Skin friction

Skin friction is measured with a rotating disc on the skin surface with constant speed and pressure. Consequently, a higher resistance of the skin results in a high skin friction [38]. A high skin friction can be caused by dry skin and/or by sticky formulations on top of the skin. In the present study, all formulations increased the skin friction at least eightfold (Fig. 6C). This suggests that all samples formed a lipid film on top of the skin. After 1 h, the effect was most pronounced for the ME and the NLC and least pronounced for the NE. Thus, supporting the TEWL and corneometer values that also suggested a more pronounced film formation for ME and NLC.

After 6 h, the skin friction was slightly increased for the untreated skin, indicating—similar to corneometer and TEWL measurements—a slight dehydration of the skin during the experiment. The skin friction of the treated skin areas was decreased when compared to the friction values after 1 h, indicating that the film forming properties of the formulations decreased over time. The decrease in skin friction was most pronounced for the oil and the ME and was almost neglectable for the NLC and the NE (Fig. 6C). Data therefore indicate a less intense substantivity for oil and ME, when compared to the NE and the NLC. The reason is the smaller particle size of the NE and the NLC, which results in an increased adhesiveness of particles. This results in less weight per particle per attaching point to the skin due to an increased surface area/particle weight ratio when compared to larger sized particles. After 6 h, the skin friction was not significantly different between ME and NE. Hence, after 6 h, the film forming effects were similar for both formulations. The NLC possessed a slightly higher skin friction than ME and NE, indicating that NLC possess the highest substantivity from all formulations tested. Results are therefore in line with the data obtained for SCT, skin hydration, and TEWL which also confirmed the presence of the invisible patch for the NLC, that resulted in an increased SCT and the most effective dermal penetration of the AI surrogate.

### Synopsis of biopharmaceutical properties

**Fig. 7:**
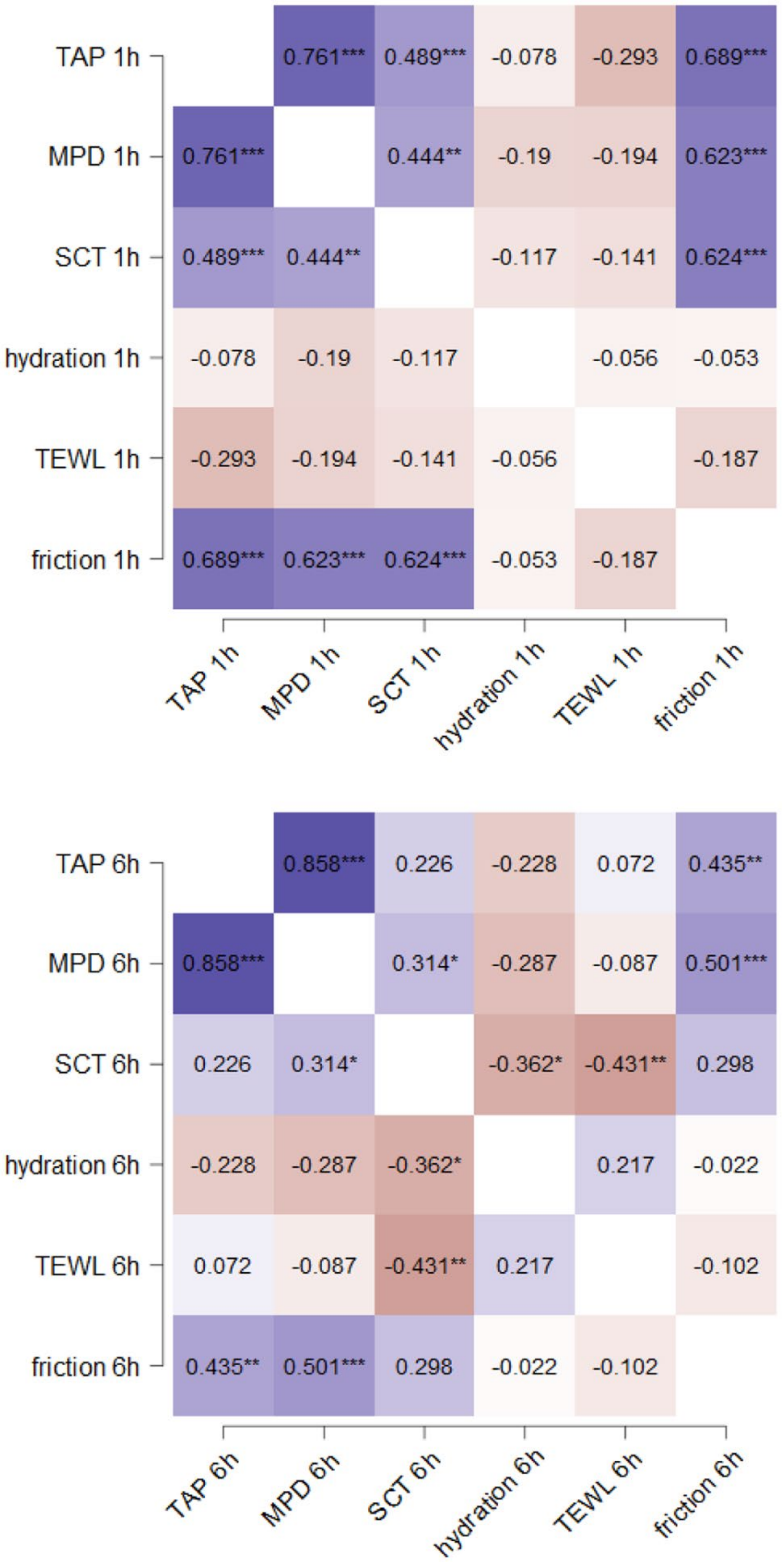
Spearman’s rank correlation coefficients for TAP, MPD, SCT, skin hydration, TEWL, and skin friction 1 h (upper) and 6 h (lower) after application of the dermal formulations. Explanations cf. text

A link between dermal penetration efficacy and skin hydration is often proposed and can also be confirmed with the data of the present study, which show a positive correlation between dermal penetration efficacy (TAP and MPD) and SCT (Fig. 7).

A high penetration efficacy was also correlated with a high skin friction and no correlation was found between penetration efficacy and skin hydration or TEWL (Fig. 7). Only after 6 h, TEWL and skin hydration became negatively correlated to the SCT. Hence, low corneometer values and low TEWL values were associated with an increased SCT, which was associated with an increased penetration efficacy. This means low TEWL values and low corneometer values can be indirectly linked via the SCT to a good dermal penetration efficacy and vice versa. Data therefore provide a significant evidence that film formation, which results in low TEWL and low corneometer values, results in a swelling of the SC, which than causes in improved dermal penetration of the AI (cf. Fig. 1).

The influence of the formulation on the chemical stability and the positive correlations between dermal penetration efficacy, SCT, and bio-physical skin parameters enable a more detailed and mechanistic understanding about the influence of the formulation on the penetration efficacy of AI into or through the skin. The results demonstrate that both chemical stability and film formation after dermal application are major prerequisites for effective dermal penetration. Data show that chemical stability of the AI must be ensured not only in the packaging container but also during the entire penetration time after dermal application. Film formation increases the skin hydration and thus the penetration efficacy. Dry skin will therefore result in poor penetration. Therefore, only long-lasting, durable films can provide an effective and long-lasting dermal penetration [37, 39–41]. Data of this study show that after dermal application of the different formulations, the NLC provided the best protection from chemical degradation for the AI surrogate and resulted in the most effective and durable film on top of the skin. Consequently, NLC led to the most effective penetration of the AI surrogate into the skin. Future studies are now needed to investigate also the influence of excipients, for example, type and amount of lipids or type and amount of surfactants on the penetration efficacy of active compounds from different formulations in more detail. This will provide more detailed knowledge and can then be the base for the development of NLC with tailor-made skin properties and penetration profiles.

## Conclusion

The proposed advantages of lipid nanoparticles, i.e., increased chemical stability for chemically labile AI, improved and longer lasting skin hydration due to the formation of an invisible patch, and an improved dermal penetration of lipophilic AI, when compared to classical formulation principles, were confirmed in this study. Data confirm that lipid nanoparticles can really combine all these beneficial properties in only one formulation, thus allowing the conclusion that NLC are a holistically superior formulation principle for dermal drug products that combines effective drug delivery and skin carrying properties (an approach that is known as “advanced corneotherapy” [42]) at the same time.

## Supplementary Information

Below is the link to the electronic supplementary material.

Supplementary file1 (DOCX 1315 KB)

## Data Availability

The datasets generated during the study are available from the corresponding author on reasonable request.

## References

[1] Müller RH, Shegokar R, Keck CM (2011). 20 years of lipid nanoparticles (SLN and NLC): present state of development and industrial applications. Curr Drug Discov Technol..

[2] Müller RH, Sinambela P, Keck CM. NLC - The invisible dermal patch for moisturizing & skin protection. Euro Cosmetics. 2013:20–2.

[3] Joshi M, Patravale V (2006). Formulation and evaluation of Nanostructured Lipid Carrier (NLC)-based gel of Valdecoxib. Drug Dev Ind Pharm..

[4] Junyaprasert VB, Teeranachaideekul V, Souto EB, Boonme P, Müller RH (2009). Q10-loaded NLC versus nanoemulsions: stability, rheology and in vitro skin permeation. Int J Pharm..

[5] Keck CM, Baisaeng N, Durand P, Prost M, Meinke MC, Müller RH (2014). Oil-enriched, ultra-small nanostructured lipid carriers (usNLC): A novel delivery system based on flip-flop structure. Int J Pharm..

[6] Keck CM, Anantaworasakul P, Patel M, Okonogi S, Singh KK, Roessner D, et al. A new concept for the treatment of atopic dermatitis: silver-nanolipid complex (sNLC). Int J Pharm. 2014;462:44–51. 10.1016/j.ijpharm.2013.12.044. 10.1016/j.ijpharm.2013.12.044 24378329

[7] Lauterbach A, Müller-Goymann CC (2015). Applications and limitations of lipid nanoparticles in dermal and transdermal drug delivery via the follicular route. Eur J Pharm Biopharm..

[8] Li X, Müller RH, Keck CM, Bou-Chacra NA (2016). Mucoadhesive dexamethasone acetate-polymyxin B sulfate cationic ocular nanoemulsion–novel combinatorial formulation concept. Die Pharmazie..

[9] Lohan SB, Bauersachs S, Ahlberg S, Baisaeng N, Keck CM, Müller RH (2015). Ultra-small lipid nanoparticles promote the penetration of coenzyme Q10 in skin cells and counteract oxidative stress. Eur J Pharm Biopharm..

[10] Lombardi Borgia S, Regehly M, Sivaramakrishnan R, Mehnert W, Korting HC, Danker K (2005). Lipid nanoparticles for skin penetration enhancement-correlation to drug localization within the particle matrix as determined by fluorescence and parelectric spectroscopy. J Control Release..

[11] Müller RH, Radtke M, Wissing SA (2002). Nanostructured lipid matrices for improved microencapsulation of drugs. Int J Pharm..

[12] Sala M, Diab R, Elaissari A, Fessi H. Lipid nanocarriers as skin drug delivery systems: Properties, mechanisms of skin interactions and medical applications. Int J Pharm 2018;535: Elsevier B.V. 10.1016/j.ijpharm.2017.10.046. 10.1016/j.ijpharm.2017.10.046 29111097

[13] Schafer-Korting M, Mehnert W, Korting HC (2007). Lipid nanoparticles for improved topical application of drugs for skin diseases. Adv Drug Deliv Rev..

[14] Newton AMJ, Kaur S. Solid lipid nanoparticles for skin and drug delivery. In: Nanoarchitectonics in Biomedicine: Elsevier; 2019. p. 295–334. 10.1016/B978-0-12-816200-2.00015-3.

[15] Muchow M, Schmitz EI, Despatova N, Maincent P, Müller RH (2009). Omega-3 fatty acids-loaded lipid nanoparticles for patient-convenient oral bioavailability enhancement. Die Pharmazie..

[16] Keck CM (2010). Particle size analysis of nanocrystals: improved analysis method. Int J Pharm..

[17] Keck CM, Müller RH (2008). Size analysis of submicron particles by laser diffractometry–90% of the published measurements are false. Int J Pharm..

[18] Müller RH (1996). Zetapotential und Partikeladung in der Laborpraxis - Einführung in die Theorie, praktische Meßdurchführung, Dateninterpretation.

[19] Nagelreiter C, Mahrhauser D, Wiatschka K, Skipiol S, Valenta C (2015). Importance of a suitable working protocol for tape stripping experiments on porcine ear skin: Influence of lipophilic formulations and strip adhesion impairment. Int J Pharm..

[20] Rueden CT, Schindelin J, Hiner MC, DeZonia BE, Walter AE, Arena ET, Eliceiri KW (2017). Image J2: ImageJ for the next generation of scientific image data. BMC Bioinform..

[21] Schneider CA, Rasband WS, Eliceiri KW (2012). NIH Image to ImageJ: 25 years of image analysis. Nat Methods..

[22] Pelikh O, Pinnapireddy SR, Keck CM (2021). Dermal Penetration Analysis of Curcumin in an ex vivo Porcine Ear Model Using Epifluorescence Microscopy and Digital Image Processing. Skin Pharmacol Physiol..

[23] Dinno A (2015). Nonparametric pairwise multiple comparisons in independent groups using Dunn’s test. The Stata Journal..

[24] Junginger HE (1984). Colloidal structures o f O / W creams. Pharm Weekbl Sci..

[25] Acar-Kübart S, Keck CM (2013). Laser diffractometry of nanoparticles: Frequent pitfalls & overlooked opportunities. Journal of Pharmaceutical Technology & Drug Research.

[26] Keck CM, Kobierski S, Ofori-Kwakye, K., et al. Resveratrol nanosuspensions: interaction of preservatives with nanocrystal production. Die Pharmazie. 2011:942–7. 10.1691/ph.2011.1038. 22312699

[27] Rachmawati H, Rahma A, Al Shaal L, Müller RH, Keck CM (2016). Destabilization Mechanism of Ionic Surfactant on Curcumin Nanocrystal against Electrolytes. Sci Pharm..

[28] Müller RH, Keck CM (2004). Challenges and solutions for the delivery of biotech drugs–a review of drug nanocrystal technology and lipid nanoparticles. J Biotechnol..

[29] Müller RH, Keck CM (2004). Drug delivery to the brain–realization by novel drug carriers. J Nanosci Nanotechnol..

[30] Müller RH, Petersen RD, Hommoss A, Pardeike J (2007). Nanostructured lipid carriers (NLC) in cosmetic dermal products. Adv Drug Deliv Rev..

[31] Pardeike J, Hommoss A, Müller RH (2009). Lipid nanoparticles (SLN, NLC) in cosmetic and pharmaceutical dermal products. Int J Pharm..

[32] Pardeike J, Müller RH. Coenzyme Q10-loaded NLCs: preparation, occlusive properties and penetration enhancement. Pharmaceutical Technology Europe. 2007;01 July.

[33] Radtke M. Grundlegende Untersuchung zur Arzneistoffinkorporation, -freisetzung und Struktur von SLN und NLC [PhD Thesis]; 2003.

[34] Radtke M, Müller RH. NLC^®^ - Nanostructured lipid carriers: the new generation of lipid drug carriers. NewDrugs. 2001;2:48–52.

[35] Purdon C, Smith E, Surber C, Zhang J, Maibach H. Penetration Enhancement by Skin Hydration. In: Smith EW, editor. Percutaneous penetration enhancers. 2nd ed. Boca Raton, FL: Taylor & Francis; 2006. p. 67–71. 10.1201/9781420039207.pt2.

[36] Lopes LB (2014). Overcoming the cutaneous barrier with microemulsions. Pharmaceutics..

[37] Pelikh O, Keck CM (2020). Hair Follicle Targeting and Dermal Drug Delivery with Curcumin Drug Nanocrystals-Essential Influence of Excipients. Nanomaterials.

[38] Scientific products, https://www.courage-khazaka.de/en/scientific-products/all-products. Accessed 12 Feb 2020.

[39] Olechowski F, Müller RH, Pyo SM (2019). BergaCare SmartLipids: commercial lipophilic active concentrates for improved performance of dermal products. Beilstein J Nanotechnol..

[40] Keck CM, Specht D, Brüßler J. Influence of lipid matrix composition on biopharmaceutical properties of lipid nanoparticles. J Control Release. 2021.10;338:149–63. 10.1016/j.jconrel.2021.08.016. 10.1016/j.jconrel.2021.08.016 34389366

[41] Pelikh O, Eckert RW, Pinnapireddy SR, Keck CM (2020). Hair follicle targeting with curcumin nanocrystals: Influence of the formulation properties on the penetration efficacy. J Control Release.

[42] Keck CM. Corneotherapie – Pflege und Reparatur der Haut: präzise, effektiv und nachhaltig. J Ästhet Chir. 2020:132–42. https://doi.org/10.1007/s12631-020-00227-9.

