# Supplementary material for: Are lipid nanoparticles really superior? A holistic proof of concept study
Source: Drug Deliv Transl Res. 2021 Sep 3;12(6):1433–44. doi: 10.1007/s13346-021-01021-5 (PMC9061673; doi:10.1007/s13346-021-01021-5)
Supplement: Supplementary file 1 — Supplementary file1 (DOCX 1315 KB) [file 13346_2021_1021_MOESM1_ESM.docx]

Supplementary materials

**Supplementary material section 1:**

Images were subjected to an automated threshold algorithm to eliminate the autofluorescence of the skin. The algorithm was programmed in ImageJ software and contained two macros that were run subsequently and likewise for each image. The program code of the macros applied is provided below:

***Macro 1:***

// Color Thresholder 1.52a

// Autogenerated macro, single images only!

min=newArray(3);

max=newArray(3);

filter=newArray(3);

a=getTitle();

run("RGB Stack");

run("Convert Stack to Images");

selectWindow("Red");

rename("0");

selectWindow("Green");

rename("1");

selectWindow("Blue");

rename("2");

min[0]=0;

max[0]=0;

filter[0]="stop";

min[1]=33;

max[1]=255;

filter[1]="pass";

min[2]=0;

max[2]=0;

filter[2]="stop";

for (i=0;i<3;i++){

selectWindow(""+i);

setThreshold(min[i], max[i]);

run("Convert to Mask");

if (filter[i]=="stop") run("Invert");

}

imageCalculator("AND create", "0","1");

imageCalculator("AND create", "Result of 0","2");

for (i=0;i<3;i++){

selectWindow(""+i);

close();

}

selectWindow("Result of 0");

close();

selectWindow("Result of Result of 0");

rename(a);

// Colour Thresholding-------------

**Macro 2:**

run("Invert");

**Supplementary material section 2:**

The physical stability of the formulations (macroemulsion (ME), nanoemulsion (NE) and nanostructured lipid carriers (NLC)) was assessed by using laser diffraction (LD), photon correlation spectroscopy (PCS) and light microscopy over a period of 5 weeks.

Storage conditions: room temperature and exclusion of light.

Fig. S2-1: Particle size analysis (LD and PCS) over 5 weeks. Mean values ± S.D.. Data showed an instability of the particles contained in the ME. In case of NE, the LD technique detected larger drops after 7 days. The particle sizes of the NLC remained unchanged - meaning a more effective stabilization of the of the lipid nanoparticles in comparison to the emulsion droplets.

*
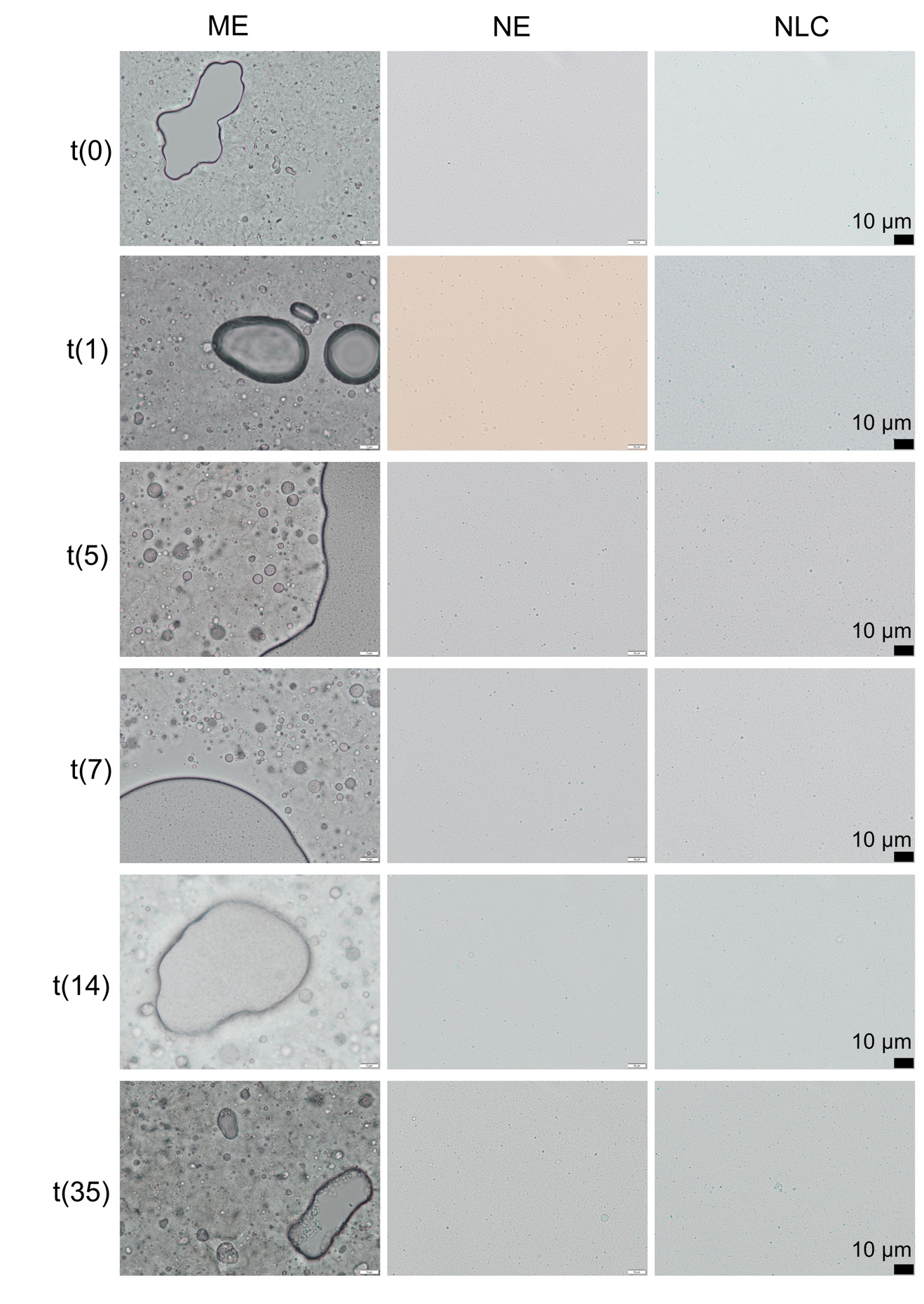
*

Fig. S2-2: Light microscopic images of the formulations. Magnification: 1,000-fold.

**Supplementary material section 3:**

Selected images of epifluorescence microscopic images (magnification: 200-fold) of skin biopsies – non-treated and treated with Dil loaded in oil, NE, NLC and ME after 1h (Fig. S3-1) and 6 h (Fig. S3-2) peneteation time.


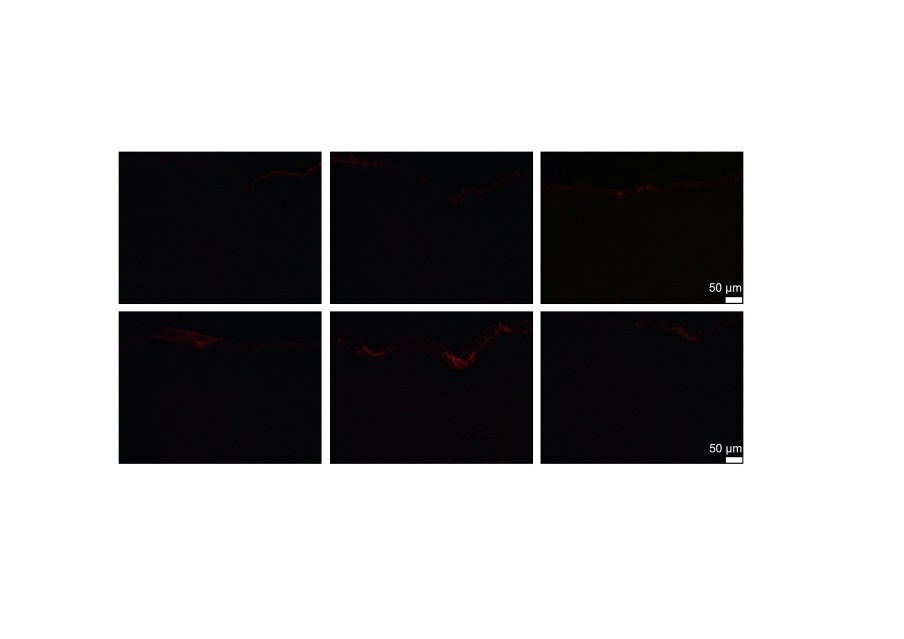


untreated skin


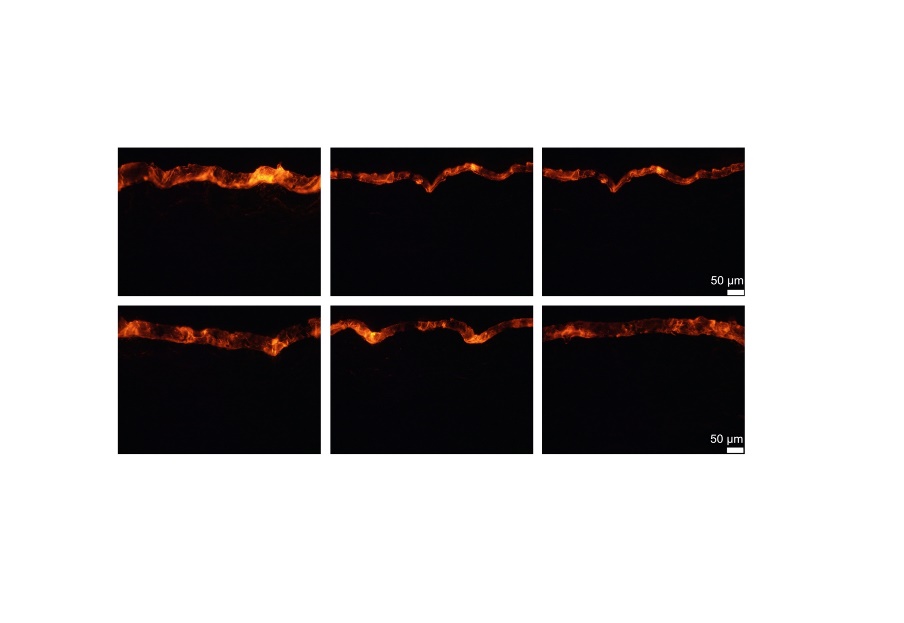


oil


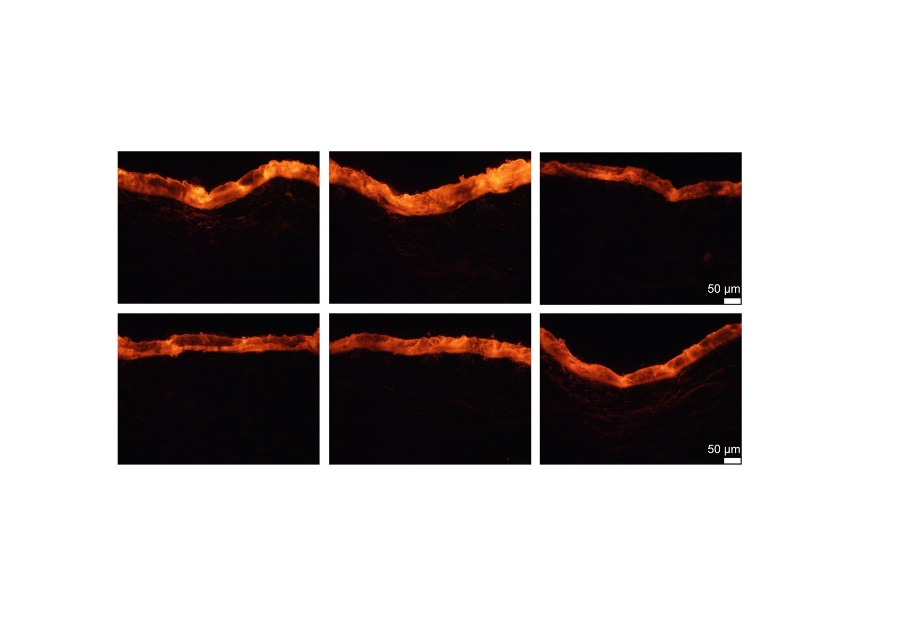


NE


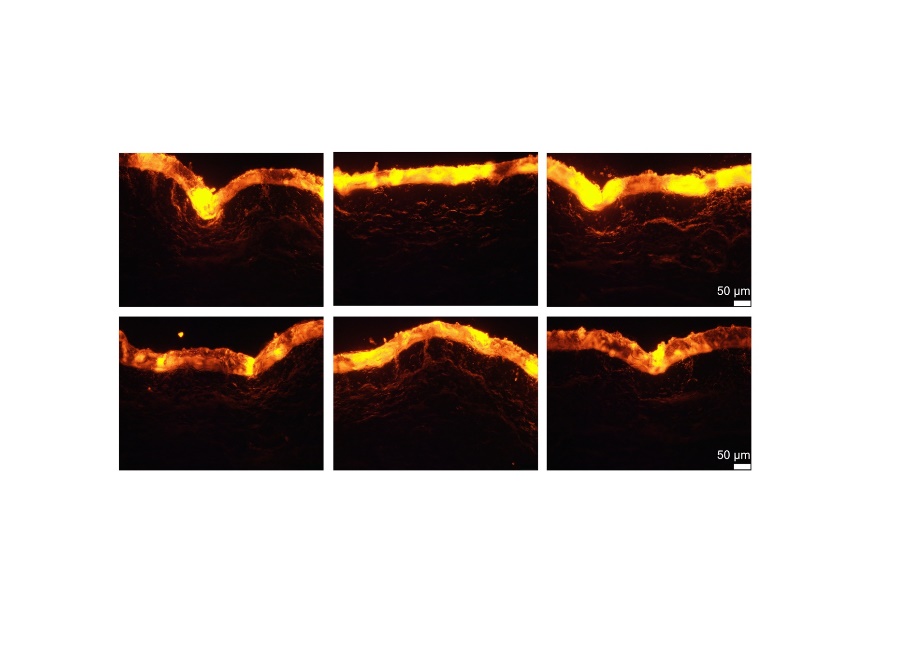


NLC


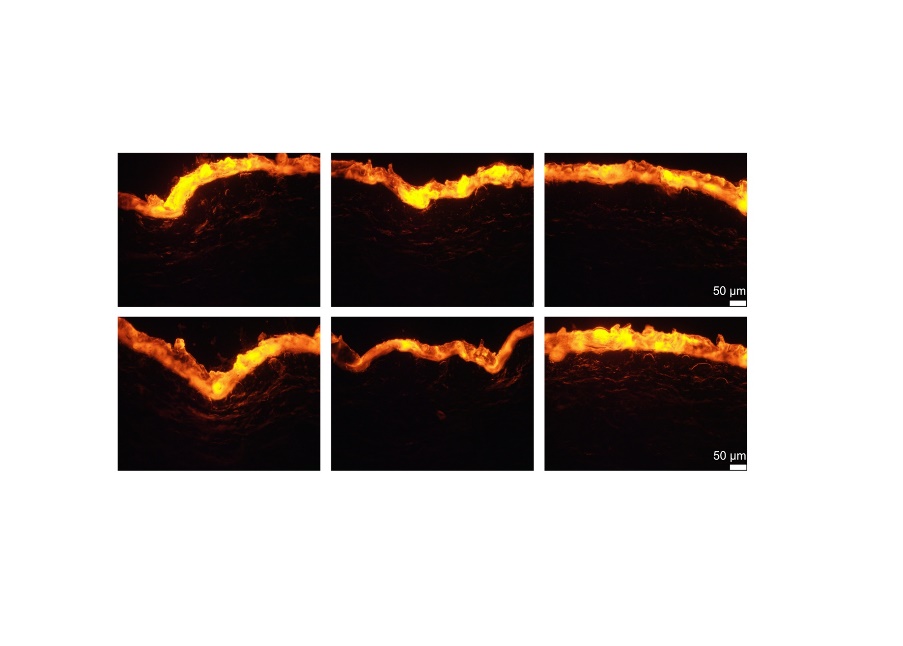


ME

***Fig. S3-1: Images of skin biopsies after 1 h penetration time.***


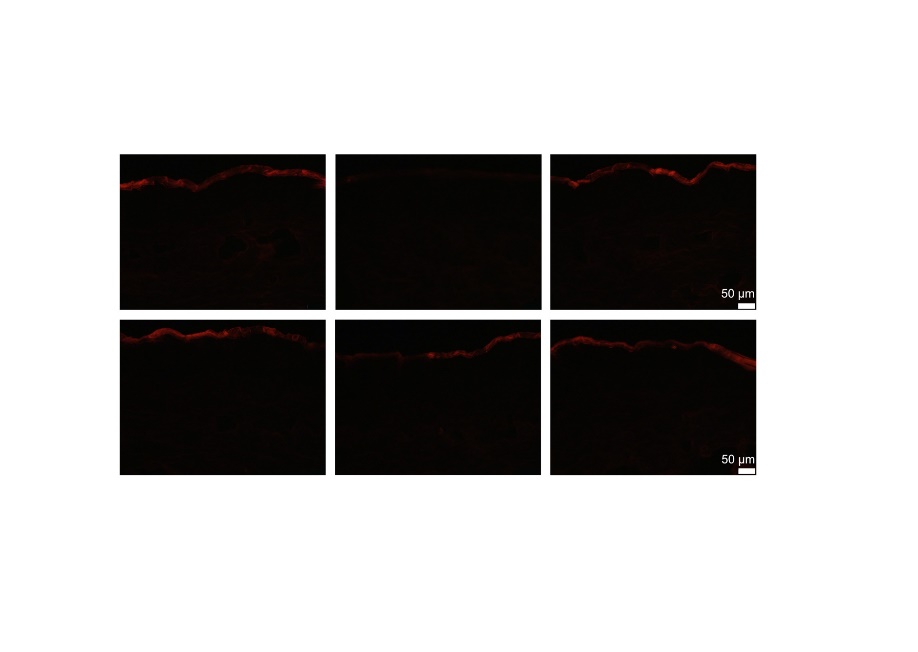


untreated skin


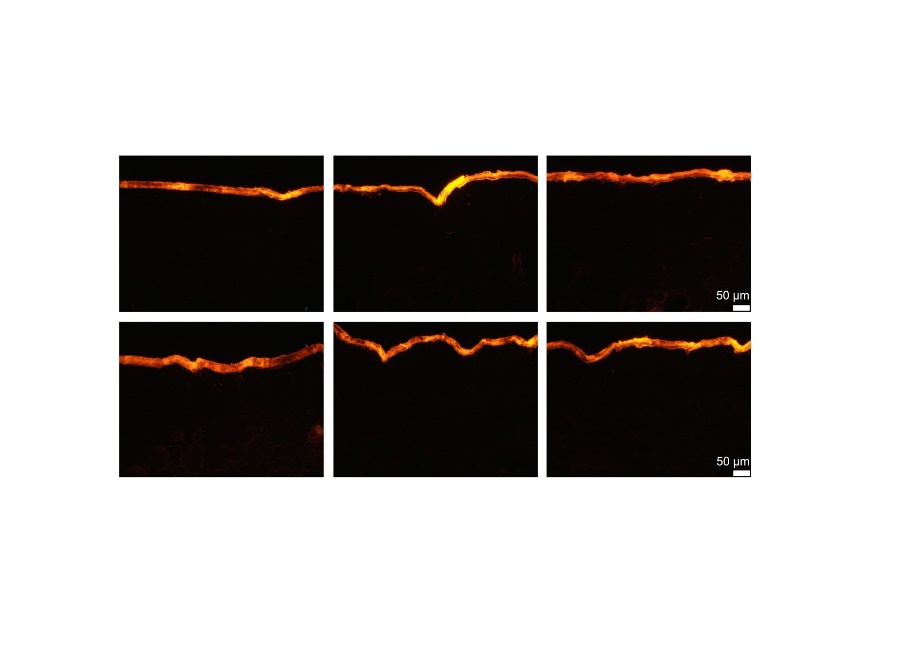


oil


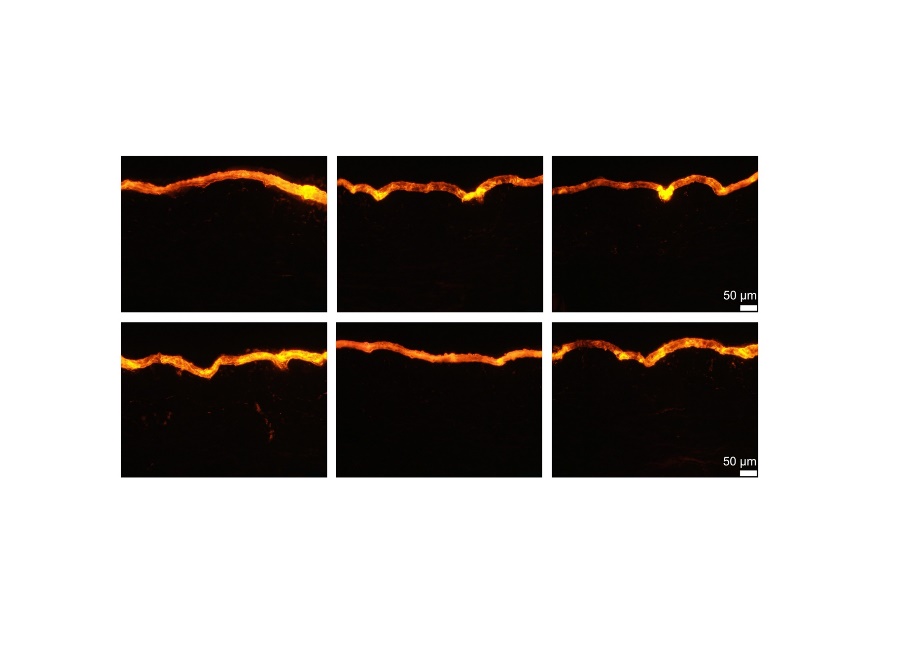


NE


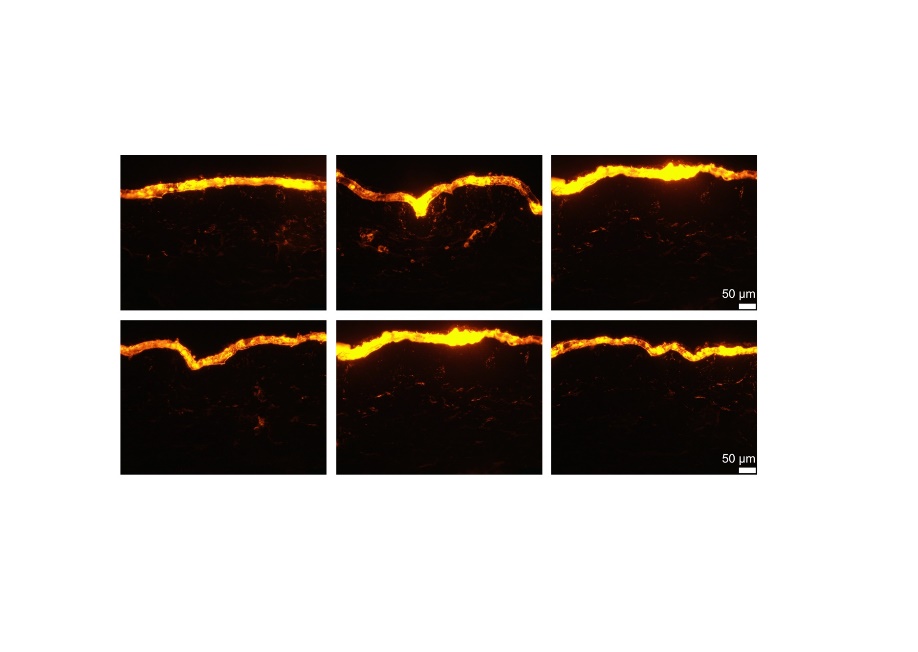


NLC


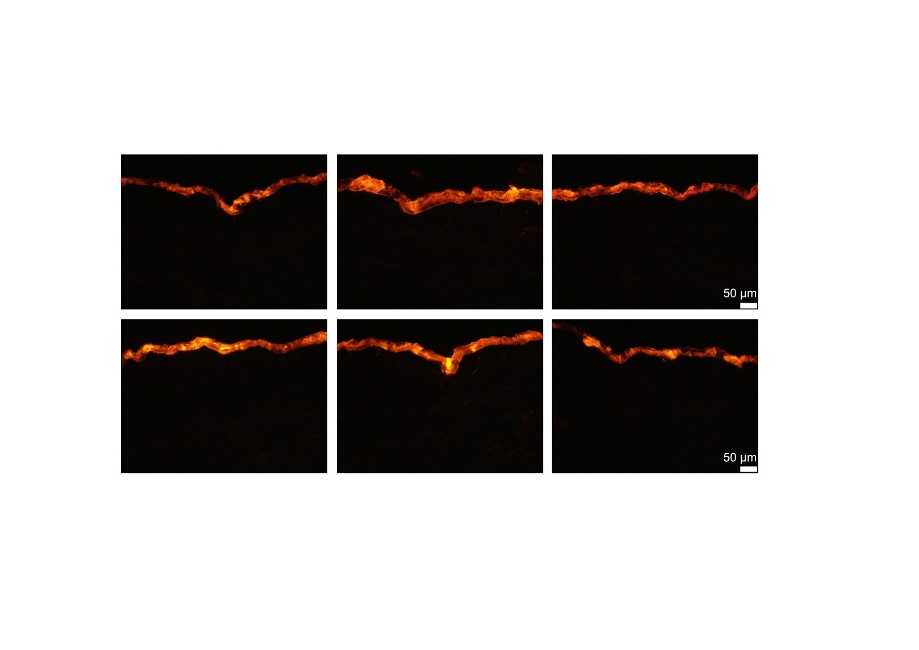


ME

***Fig. S3-2: Images of skin biopsies after 6 h penetration time.***
